# A Benchtop Evaluation of Cervical Collar Design and Strap Tension

**DOI:** 10.2147/MDER.S541594

**Published:** 2026-02-02

**Authors:** Laurence Jeppe Russell, Liudi Jiang, Davide Filingeri, Peter R Worsley

**Affiliations:** 1 School of Health Sciences, Faculty of Environmental and Life Sciences, University of Southampton, Southampton, UK; 2 Department of Mechanical Engineering, Faculty of Engineering and Physical Sciences, University of Southampton, Southampton, UK

**Keywords:** cervical collars, pressure ulcers, interface pressure

## Abstract

This article presents a novel experimental setup for objectively evaluating cervical collar design and fit by monitoring strap tension and interface pressures across the device-skin interface. This study evaluated four commercially available cervical collars, achieving repeatable findings (ICC > 0.93) that were sensitive to differences between collars and across strap tensions. For each collar, there was a significant relationship (r > 0.4, p < 0.005) between strap tension and interface pressure values, with pressures exceeding 100 mmHg for high strap tensions. Differences in the distribution of pressures and the relationship between strap tension and interface pressures highlight the need for these objective measures of design and fit for cervical collars to evaluate new and existing designs.

## Introduction

Cervical collars are routinely used to support head alignment and restrict movement in the cervical spine. They are used to manage a range of conditions. In the care of trauma patients with suspected spinal injury, collars are used to restrict movement, preventing further damage to the spinal cord.^1^ In the care of cancer patients, cervical collars are used to support the neck after surgical intervention, aiding the rehabilitation process.^2^ In cases of drop-neck or motor neuron disease, the cervical collar provides support to help an individual with activities of daily living.^3^ A variety of designs are used to meet the range of needs, from the most severe motion restriction with extrication collars to soft foam collars that provide gentle support. Depending on the design and purpose, varying loads can be placed on the underlying skin and soft tissue, which include pressure, temperature, and humidity.^4^

Collars generally comprise a stiff component that supports the spine and a softer padding for comfort. The amount of padding and rigidity of the collars varies between designs and their primary purpose. Off-the-shelf cervical collars usually have some amount of adjustability or are sold in a variety of sizes. Built-in vertical adjustments and the use of circumferential straps allow for both height and width adjustments. This attempts to account for the large variability in head, neck, and shoulder geometries. Effective collar function is highly dependent on fit and application.^5^ There is also an underlying trade-off in collar tension adjusted through the straps between restriction of the cervical spine, and device-skin interface pressures and comfort.^4^

Mechanical loading on the skin from external medical devices in the form of pressure and shear presents a significant risk of damage to the skin and underlying soft tissues.^6^ When these mechanical loads exceed the tolerance of the tissue, they can form pressure ulcers (PU), also termed pressure injury.^7^ The stiff supports and strapping of medical devices often lead to localised areas of pressure and shear at the device-skin interface.^8^ In vivo experimental studies have shown that different collar designs introduce varied interface pressure, indicating variable PU risk.^4^,^9–12^ While increased strap tension in cervical collars is linked to less spinal movement, corresponding increases in inflammatory response and temperatures are observed at the interface.^12^ Both are associated with an increased risk of pressure ulcers.^13^ Additionally, ill-fitting collars, resulting from design or application, can lead to pressure and shear concentrations that further increase the risk of pressure ulcers. Depending on the designs, these interface pressure and shear conditions will vary in magnitude and location. Therefore, monitoring the effects of design choices on the interface pressures is essential when optimising collar designs to minimise the risk of pressure ulcers.

Through physical modelling we can gain insight into the biomechanics of medical device-skin interfaces. Physical modelling has often been used to provide insights into interface biomechanics. Lei, Yang and Zhuang^14^ measured pressures across the device skin interface of an N95 respirator mask to better understand device fit. Sparks, Vavalle, Kasting, Long, Tanaka, Sanger, Schnell and Conner-Kerr^15^ investigate the use of silicone as a model of soft tissue mechanics to estimate risk of deep tissue injuries. Similarly, Rankin, Steer, Paton, Mavrogordato, Marter, Worsley, Browne and Dickinson^16^ use physical modelling to estimate the soft tissue strains of a residuum in a prosthetic socket. Physical models can be designed with varying levels of complexity to meet the needs of the research and answer specific questions. However, to date, physical models have not been developed to simulate the effects of collar design and strap tension.

The tension induced by tightened straps is an essential factor to consider in the fit of cervical collars.^12^ This needs to be considered in conjunction with device effectiveness and interface pressures. Manufacturers’ guidelines regarding the tightening of cervical collars are often ambiguous, citing a comfortable tension whilst restricting movement. This study aims to develop an objective methodology for reliably and repeatably measuring cervical collar interface pressures across strap tensions. To achieve this, our objectives were to set up a benchtop test to simultaneously measure tension in the collar straps and pressure at the device skin interface while applying collars to a physical phantom head model.

## Methods

### Head Model

**Figure 1:**
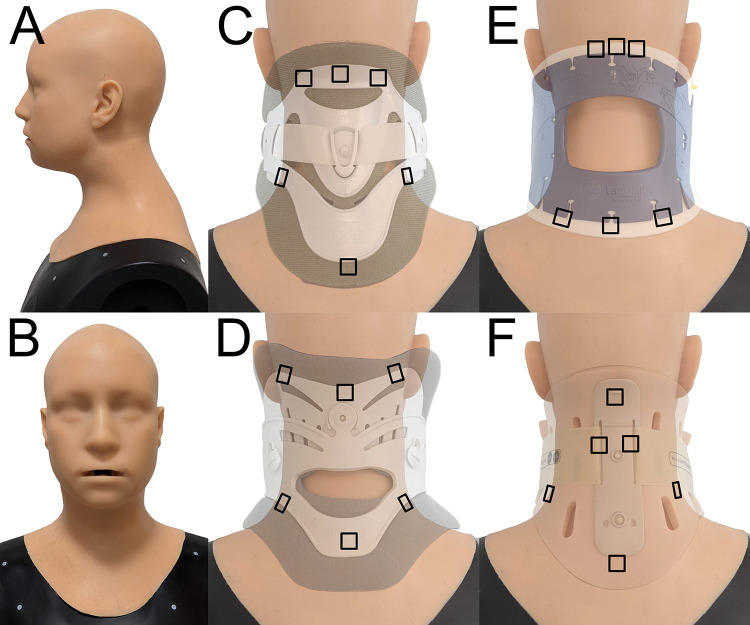
Physical phantom head model (**A**) lateral and (**B**) front views and sensor locations represented with black squares for (**C**) Aspen Vista, (**D**) Miami J, (**E**) Stiffneck, and (**F**) Philadelphia collars.

A physical phantom head model (Crawley Creatures Ltd, UK) was used to model the human head (Figure 1A: lateral view, Figure 1B: front view). Its outer geometry is based on the NIOSH medium headform:^17^ a phantom mean head shape created from surface scans of the US workforce. The model comprises a carbon composite shell with a soft elastomer skin layer covering the head, neck, and a portion of the shoulders. The elastomer skin has a manufacturer-reported shore hardness of 00–30, which is within the expected range of 00–00 to 00–40 for healthy skin.^18^

### Collars

This study investigated four commercially available cervical collars; Aspen Vista collar (Aspen Medical Products, Irvine, CA, USA), Philadelphia adjustable tracheotomy collar (Össur UK Ltd, Stockport, UK), Miami J standard collar (Össur UK Ltd, Stockport, UK), and Stiffneck Select collar (Laerdal Medical Ltd, Orpington, UK). The Aspen Vista (Figure 1C), Miami J (Figure 1D), and Philadelphia (Figure 1F) collars have separate front and rear sections attached with hook and loop straps. The Stiffneck Select collar comprises one continuous piece that wraps around the neck and secures with a hook and loop strap (Figure 1E). The collars represent a range of specifications and cost.

### Strap Tensioning Device

**Figure 2:**
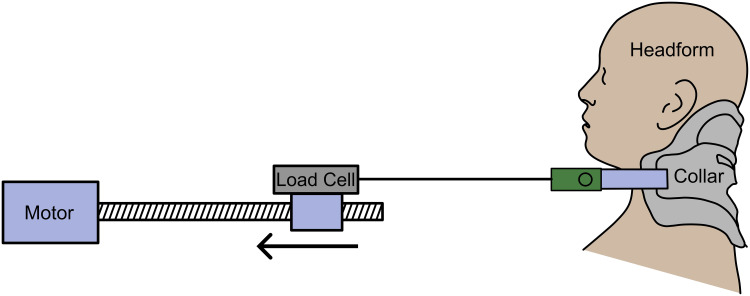
Experimental set-up with linear actuator, load cell, phantom physical head model, and cervical collar.

A servomotor-driven linear actuator with integrated load cell was used to apply tension to the straps of the collar while measuring the tension and displacement of the straps (Figure 2). The custom device included a load cell (Loadstar, model RAS1-250S-S) with USB interface (Loadstar, DI-1000U) and displacement driver to perform prescribed increments of strap tension. The force and displacement data were continuously logged to a user interface (Loadstar, SensorVUE) via a laptop (10Hz). Collar straps were attached to the device via plastic clamps and a metal cable. Where the Stiffneck collar lacks a strap on one side, a length of cord was fixed to the collar to mirror the action of the strap on the other side.

### Interface Pressure

The Tactilus Freeform pressure monitoring system (Sensor Products Inc, US) was used with their ultrasensitive V Series pressure sensors to capture the interface pressures during periods of collar application, with an operating range of 0–258 mmHg. These 1×1 cm sensors have also been used in a previous study to measure the device-skin interface pressure of N95 respirators, where the accuracy of the sensors was measured and reported to be ±3%.^14^ Data were recorded with a sampling rate of ~50 Hz. Six sensors were strategically placed at contact loading areas across the occiput, back, and shoulders. The areas of contact varied between collars, depending on their geometry. Therefore, a specific set of sensor locations was defined for each collar (Figure 1). For all collars, three sensors were placed along where the top edge of the collar contacted the head (upper cervical area) and three were placed along the bottom edge (shoulders).

### Test Protocol

**Figure 3:**
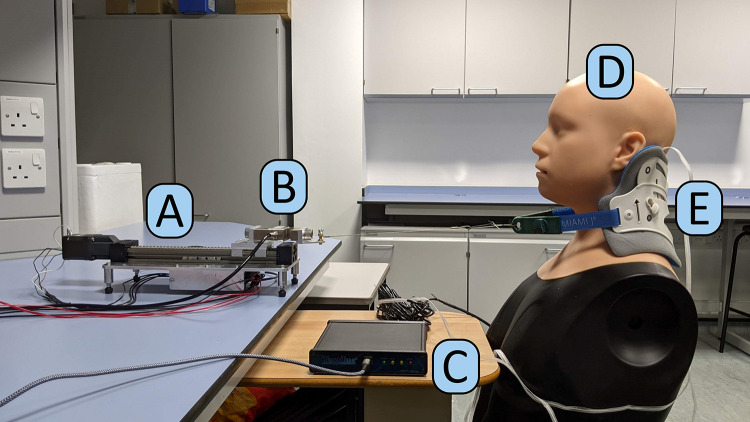
Exemplar experimental set-up with (**A**) linear actuator, (**B**) load cell, (**C**) pressure sensing system, (**D**) physical head model, and (**E**) collar.

An in-vitro bench testing methodology was developed and used to evaluate four collar designs. The head was fixed and positioned such that the collar straps were level and aligned with the displacement rig (Figure 3). The custom tensioning device applied tension to the rear portion of each of the collars on a physical phantom head model via a metal cable (Figure 3). Collar tension and interface pressures were recorded simultaneously throughout the testing. The automated testing methodology consisted of applying displacement steps of 2 mm to the straps with a 60-second pause between each step. This accommodated the viscoelastic response and ensured a period of static strap tension. Displacement steps were continued until the load cell measured a tension of above 50 N, at which point the load was released. Each collar was tested three times. A period of ten minutes was allowed for the foam material to recover between each test.

Additionally, the collars were applied manually to the head without the automated tensioning device at three self-determined tensions: low, medium, and high. This was performed by the same researcher using perceived tensioning of the straps. This provides a means of comparison for the automated tension tests. Due to sensor damage, the manual tests were limited to the Miami J, Aspen Vista, and Philadelphia collars.

### Data Processing and Statistics

Data were processed with Python (version 3.9), pandas, SciPY, and matplotlib libraries. Pressure and tension data were synchronised and resampled at 10 Hz. Local minima were identified in the strap tension data, indicating the moment before the following tension step. Linear regression analysis was performed on this data across the combined upper sensors and the combined shoulder sensors, respectively, to evaluate the tension-pressure relationships. Further quadratic interpolation was conducted to get corresponding data points at 2.5 N increments of tension between 5 N and 30 N for comparison between collars. Intraclass correlation coefficients (ICCs) were calculated for absolute agreement across three repeated measures across the interpolated strap tension increments, with excellent, good, and moderate correlations defined as ICC values of > 0.9, 0.75–0.9, and 0.5–0.75, respectively.^19^ Cumulative interface pressures across all sensor locations were used to compare manual and automatic tests. The corresponding tensions were then defined for low, medium, and high manual collar application.

## Results

### Relationship Between Collar Strap Tension and Interface Pressure

**Figure 4:**
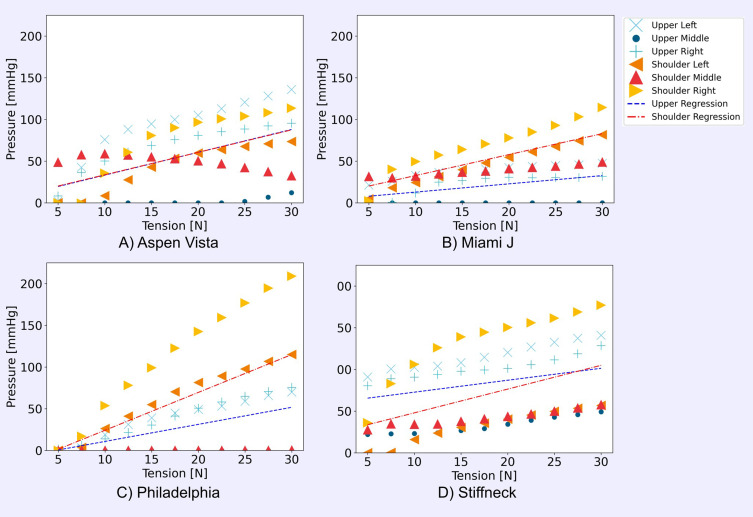
Mean pressure to strap tension relationship for (**A**) Aspen Vista collar, (**B**) Miami J collar, (**C**) Philadelphia collar, and (**D**) Stiffneck collar.

Interface pressure generally increased with greater strap tension, though the rate of increase differed between collars and sensor locations (Figure 4A–D). Despite several of the pressure readings showing somewhat non-linear responses to strap tension, across all the collars, there were moderate to strong positive linear correlations between strap tension and pressure (Table 1). This varied considerably between collars and between the upper and shoulder regions of the interface, with the upper regions generally having higher gradients. The Stiffneck collar had the highest interface pressures of all the designs, especially at low strap tensions (Figure 4D). This was confirmed by the high y-intercept from the linear regression of the mean interface pressures (Table 1), especially across the upper sensors (58.4 mmHg). Though initially low, interface pressure in the Philadelphia collar increased with a high gradient following increased strap tension, resulting in the highest values ~200 mmHg at the 30 N tension. The Miami J collar had the lowest gradient of 1.0 and 2.5 mmHg/N (Table 1), across the upper and shoulder regions respectively, indicating that increases in tension had a less significant impact on the interface pressure.

**Table 1:** Linear Regression Statistics of Interface Pressure Across Upper and Shoulder Sensors for Each Collar Design, with p-value of the Linear Regression

		Aspen Vista	Miami J	Philadelphia	Stiffneck
**Upper**	Gradient [mmHg/N]	2.7	1.0	2.0	1.4
	Y intercept [mmHg]	5.9	3.0	−9.7	58.4
	Pearsons’ R-value	0.49	0.42	0.73	0.28
	p-value	< 0.0001	< 0.0001	< 0.0001	< 0.0030
**Shoulders**	Gradient [mmHg/N]	2.7	2.5	4.5	2.9
	Y intercept [mmHg]	6.7	7.6	−21.2	19.4
	Pearsons’ R-value	0.64	0.77	0.59	0.43
	p-value	< 0.0001	< 0.0001	< 0.0001	< 0.0001

### Manual vs Automated Collar Application Interface Pressure

**Figure 5:**
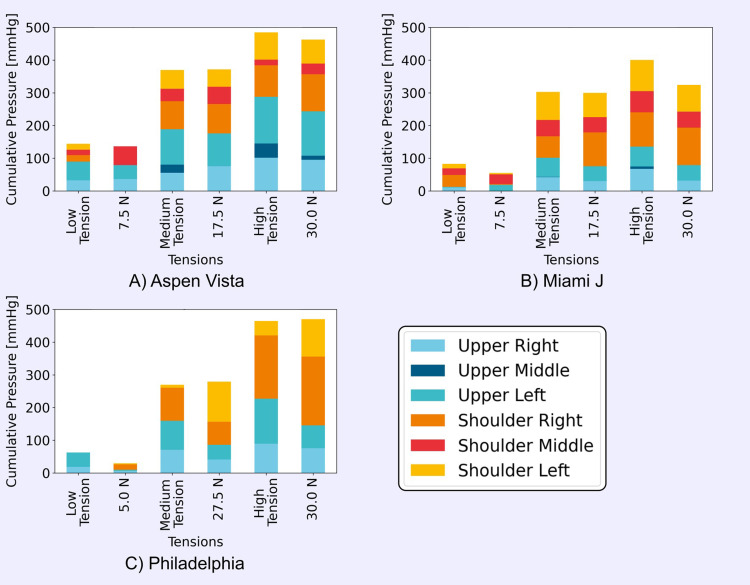
Stack plots comparing low, medium and high manual strap tensions to equivalent tensions in the automated tests across six sensor locations for the (**A**) Aspen Vista, (**B**) Miami J, and (**C**) Philadelphia collars.

A similar relationship between strap tension and interface pressure was observed when applying the collars to the head model manually (Figure 5). Figure 5 compares the cumulative pressures across the six sensors between the manually applied low, medium, and high tensions to the closest corresponding tensions from the automated collar application tests. Low, medium, and high tensions correlated with 5–7.5 N, 17.5–27.5 N, and 30 N of strap force, respectively. The distributions of pressures across the sensor locations were similar between the automated and manual tests. It is clear from these plots that the distributions of pressure across the different sensor locations are different between collar designs. The Aspen Vista collar had high pressures across the upper cervical region, while the Miami J collar had higher values across the shoulders.

### Reliability of Collar Testing

High ICC values (greater than 0.9) are seen across all collars and sensor locations (Table 2). Alongside this, standard error of measurement (SEM) values were low, ranging between 0.2 and 3.0 mmHg. There were three instances where pressure sensors recorded zero pressure across the whole test: the middle shoulder and middle occiput sensors for the Philadelphia collar, and the middle shoulder sensor for the Miami J collar. Correlation coefficients could not be calculated for these three readings.

**Table 2:** Table of Correlation Coefficients (ICC) and 95% Confidence Intervals (CI) for Each Collar and Location for Strap Tensions Between 5 and 30 N

Location		Correlation Coefficient (95% Confidence Interval)			
		Aspen Vista	Miami J	Philadelphia	Stiffneck
**Upper Cervical**	Middle	0.975 ([0.921, 0.992])	0.982 ([0.813, 0.996])	N/A	0.981 ([0.771, 0.996])
	Right	0.998 ([0.994,** **0.999])	0.978 ([0.659, 0.996])	0.997 ([0.948, 0.999])	0.998 ([0.979, 1.000])
	Left	0.983 ([0.864, 0.996])	0.927 ([0.357, 0.985])	0.976 ([0.722,** **0.995])	0.997 ([0.983, 0.999])
**Shoulder**	Middle	0.958 ([0.885, 0.988])	N/A	N/A	0.971 ([0.782, 0.993])
	Right	0.997 ([0.990, 0.999])	0.972 ([0.851, 0.993])	0.993 ([0.920, 0.999])	0.956 ([0.547, 0.991])
	Left	0.991 ([0.975, 0.997])	0.961 ([0.805, 0.990])	0.998 ([0.994, 0.999])	0.984 ([0.725, 0.997])

## Discussion

This study proposes a novel in vitro modelling methodology for objectively evaluating the effect of strap tension when applying cervical collars. By evaluating the relationship between cervical collar-skin interface pressures and strap tension, the risk of skin damage from ill-fitting collars can be estimated. This new benchtop test was sensitive to detect differences in collar design and corresponding interface pressures resulting from strap tensioning. Additionally, the repeatability of the interface pressure measurements was high (ICC > 0.9) across most collars and sensor locations. Some sensors recorded no pressure, where it was observed that device-skin contact was not achieved. Interface pressure values exceeded 100 mmHg under the highest strap tensions in several collars, which may impact the skin and subdermal tissues if an individual were to wear a collar for prolonged periods.^20^ This physical model methodology provides an objective means to test different collar designs under varying strap tensions to optimise collar geometry and material properties for current and future designs.

Some strong positive linear correlations existed between strap tension and interface pressure (r 0.28–0.77, p < 0.0001). However, there was a high degree of inter-collar variability. Some collars showed a nonlinear pressure response to the added tension, indicating a redistribution of pressure and growth in the contact area. Other collars showed a more linear relationship, indicating the contact was more consistent across the tensions. The gradient of the strap tension and interface pressure relationship differed between collars by a factor of 2.7 across the upper region (Miami J: 1.0 mmHg/N, Aspen Vista: 2.7 mmHg/N) and 1.8 for the shoulder region (Miami J: 2.5 mmHg/N, Philadelphia: 4.5 mmHg/N). The benchtop method was sensitive to detect changes in collar designs under varying tensions. With a steeper gradient comes an increased risk of overtightening, where small increases in tension may result in high interface pressures above a tolerable range.^21^ Interface pressures across the Stiffneck collar were high even at low strap tensions. This highlights the risk of pressure ulcers that these collars present. In vivo studies have also found significant differences in interface pressure between collar designs.^4^,^9^,^10^,^22^ Trends regarding the collars that expose the skin to the highest and lowest pressures are consistent with this study. Plaisier, Gabram, Schwartz and Jacobs^22^ found highest pressures for the Stiffneck (49 mmHg) compared to the Philadelphia (21 mmHg) and Miami J (13 mmHg) at the occiput. Differences in measurement techniques, sensor types, and sensor locations make comparisons of absolute values difficult.

There was also a large variability in the interface pressures across the different sensor locations for each collar. An uneven distribution of interface pressures across the contact might indicate a suboptimal condition with pressure concentrations. Pressure concentrations, especially over bony prominences like the occiput, increase the skin’s risk of ulceration.^21^,^23^ Both bony prominences and localised pressure concentrations introduce a combination of shear and normal stresses and strains within the soft tissue that compromise tissue health.^6^ In this study, collars with more flexible materials and softer foam padding were better at distributing pressure across the contact locations (eg Aspen Vista, Miami J). In contrast, collars with stiffer materials had higher pressure peaks and uneven distribution of pressure across the sensor locations (eg Stiffneck, Philadelphia). The softer foams are more compliant to the shape of the user and therefore better distribute pressure across the interface. The relationship between strap tension and interface pressures differed across the measurement sites. Differences in the relationship between tension and interface pressures indicate a redistribution of pressures across the interface, especially where the response is non-linear. This could have resulted from deformation of the collar outer material or a viscoelastic response from the foam inner surface. Further work is needed to understand how interface pressures are distributed across a range of tensions and head shapes and sizes. Furthermore, consideration must be given to what an optimal pressure distribution looks like.

The incidence of pressure ulcers indicates that the occiput is a high-risk location.^24^ In vivo literature supports this with high interface pressures.^4^,^9^,^10^ This study found high pressures at the left and right aspects of the occiput for some collar designs, eg > 100 mmHg for Stiffneck. The lower central occiput value could have been attributed to the experimental setup; unlike other studies that examine pressure in a supine position, this was an upright test in which body weight and support surface are not considered. This study demonstrates that there is a trade-off between effective immobilisation and optimal skin health. Optimisation of collar designs should consider effective immobilisation and skin health in combination.

## Limitations

This methodology only measured a few discrete pressure points on a few collar designs on a single physical phantom head model. Even with careful placement of pressure sensors, conclusions about pressure distribution are tenuous. Higher-resolution sensor arrays or finite element models could give a better understanding of device-skin interface mechanics. Due to the differences in contact between collar designs, direct like-for-like comparisons are also limited. The head model is characteristic of a 50th centile male head shape. High inter- and intra-gender head shape variability limits the generalisability of the findings presented here.^25^ Furthermore, though close, the material properties of the head models’ skin do not accurately represent human skin and will influence the interface mechanics. Further work is needed to fully understand the effects of head size, shape, and skin properties on the distribution of pressures across the device skin interface. Parameterised computational modelling could provide insights into the influence of these factors.^26^ This study did not study the effectiveness of cervical immobilisation. It is evident that effective immobilisation goes hand in hand with collar tightness.^12^ Additionally, this study only considered quasistatic loading. This does not take into consideration small variations in user movements or collar fitting. This also neglects the long-term effects of wear of the collar.

## Conclusions

The proposed test method has been shown to reliably differentiate collar designs and the effects of strap tension on interface pressure. Indeed, the tightness of collar straps has a clear impact on collar-skin interface pressures. However, this is highly dependent on collar design and locations across the contact interface. It is, therefore, important that special considerations are made when tightening collar straps to optimise skin health. Irrespective of collar design, pressure values exceeded 100 mmHg in different locations. Therefore, there is scope to improve the design of collars to provide effective cervical support whilst minimising the risk of skin damage. This method could serve as a bench testing platform to support collar innovation as well as training for healthcare professionals to help them understand the balance between strap tension and skin health.

## Data Sharing Statement

The data will be available upon publication at the University of Southampton data repository, PURE.
